# Supplementary material for: New black indium oxide—tandem photothermal CO2-H2 methanol selective catalyst
Source: Nat Commun. 2022 Mar 21;13:1512. doi: 10.1038/s41467-022-29222-7 (PMC8938479; doi:10.1038/s41467-022-29222-7)
Supplement: Supplementary file 1 — Supplementary Information [file 41467_2022_29222_MOESM1_ESM.pdf]

Supplementary Information

New Black Indium Oxide – Tandem Photothermal CO_2-H_2 Methanol Selective Catalyst

Zeshu Zhang, Chengliang Mao*, Débora Motta Meira, Paul N. Duchesne, Athanasios A. Tountas, Zhao Li, Chenyue Qiu, Sanli Tang, Rui Song, Xue Ding, Junchuan Sun, Jiangfan Yu, Jane Y. Howe, Wenguang Tu, Lu Wang*, Geoffrey A. Ozin*

The photothermal advantage for CO

In **Figure 2d**, the E_a also represents the catalytic mechanism for the whole reaction. Because E_a for dark and light are very close to each other, we assume that the black indium oxide only has the same reaction mechanism for RWGS with and without light, which means it is a photothermal/thermal process. Then the obtained CO rate with light can be used to calculate the temperature via the Arrhenius plot (dark).

$$y = -17.004x + 34.957 \text{ (dark)}$$

when y = 3.433 (ln(CO rate) at 250 °C with light)

$$x = 1.8539 \Rightarrow x = 1000/T \Rightarrow T = 539.4 \text{ K} \Rightarrow T = 266.4 \text{ °C}$$

As a result, at 250 °C, the black indium oxide has a local temperature about 266 °C, given a 16 °C photothermal advantage (CO rate of 19.7 umol g^{-1} h^{-1}).

The photothermal advantage for methanol

In **Figure 2f**, the different temperature ranges with and without light also imply an about 20 °C photothermal advantage, which agrees well with the previous estimation of local temperature (about 16 °C). A similar method was applied to MeOH's Arrhenius plot for low temperature.

$$y = -20.218x + 39.418 \text{ (Dark-low)}$$

when y = 1.9727 (ln(MeOH rate) at 250 °C with light)

$$x = 1.8521 \Rightarrow x = 1000/T \Rightarrow T = 539.93 \text{ K} \Rightarrow T = 266.92 \text{ °C}$$

The estimated local temperature agrees well with the previous estimation (about 16 °C photothermal advantage, MeOH rate of 5.14 umol g^{-1} h^{-1}). Therefore, the actual photothermal contribution for black indium oxide is about 16 °C under light conditions (lower than 250 °C).

Estimation of x, y, and z value of $H_zIn_2O_{3-x}(OH)_y$

According to charge balance in **$H_zIn_2O_{3-x}(OH)_y$** sample, then the equation is obtained:

$$2 * 3 = z + (3 - x) * 2 + y \Rightarrow z + y = 2x \qquad (1)$$

Moreover, the ^1H solid-state MAS NMR spectra of S2 shows that the ratio of hydride/hydroxyl (**Supplementary Figure 8**), following equation:

$$y = 334.5z \qquad (2)$$

Then above equation (2) is substituted equation (1), we can obtain the equation:

$$y = 1.994x \qquad (3)$$

Based on the fitting profile of core level O 1s XPS spectra, the concentration of lattice oxygen and hydroxyl over the surface S2 are 43.88% and 11.25%, respectively.

$$\frac{3-x}{Vo+(3-x)+y} = 43.88\% \qquad (4)$$

$$\frac{y}{Vo+(3-x)+y} = 11.25\% \qquad (5)$$

Combined equation (2), (3), (4) and (5), the value of x, y, z were obtained

x=0.37; y = 0.74; z = 0.0022

Therefore, the S2 chemical formula is $H_{0.0022}In_2O_{2.63}(OH)_{0.74}$.

The above results show that some hydrides remain on the precursor ($In_2O_{3-a}H_b$) surface even after the water rinsing process.

Supplementary Table 1. Quantitative analysis for surface O from the O*1s* XPS.

	S1	S2	S2-used
In-O	75.77%	43.88%	47.46%
[O]	24.23%	44.87%	43.02%
In-OH	0	11.25%	9.52%

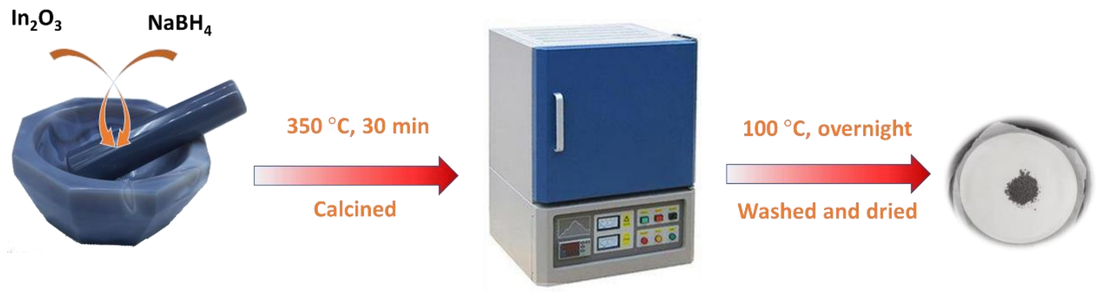

Supplementary Figure 1. The synthesis scheme of black In_2O_3 from the commercial In_2O_3. Commercially In_2O_3 and $NaBH_4$ (weight ratio 1:1.5) were added into an agate mortar. The mixture was then transferred into a muffle furnace to be calcined at 350 °C for 30 minutes (In_2O_3 + $NaBH_4$ → $In_2O_{3-a}H_b$). Finally, the black In_2O_3 was obtained in an oven at 100 °C overnight.

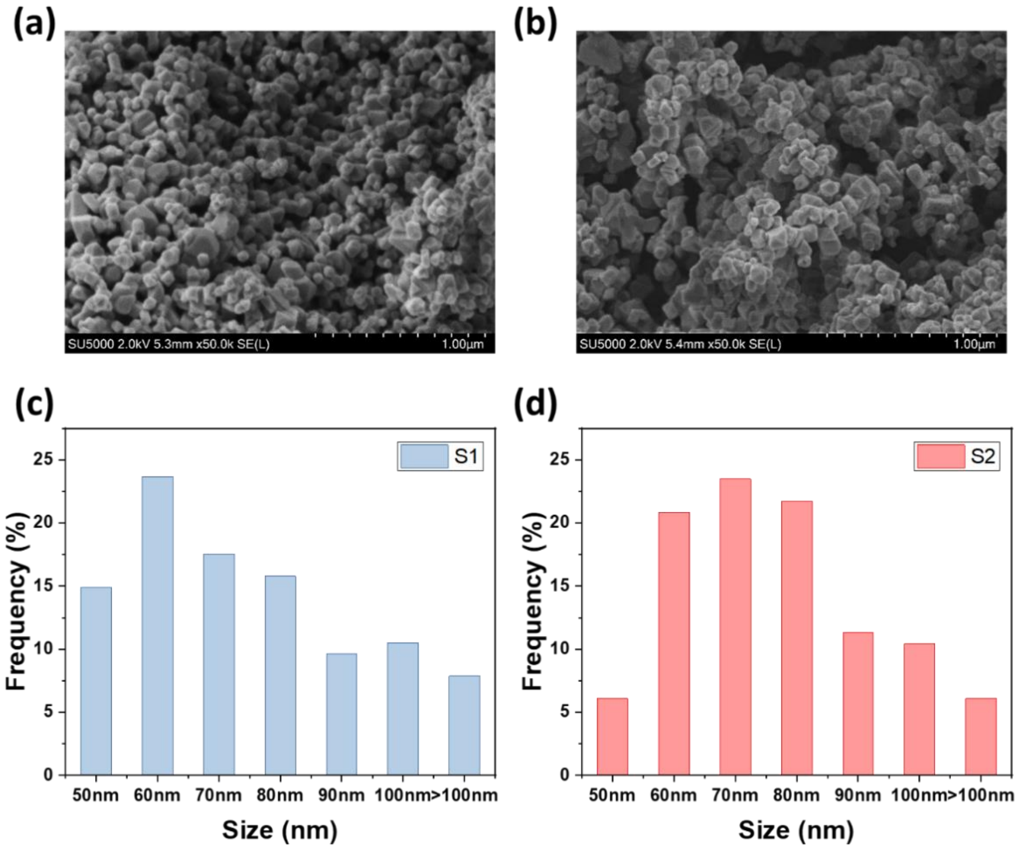

Supplementary Figure 2. SEM images and the sizes distribution. (a) SEM images of S1 and (b) S2, and the size distribution of (c) S1 and (d) S2.

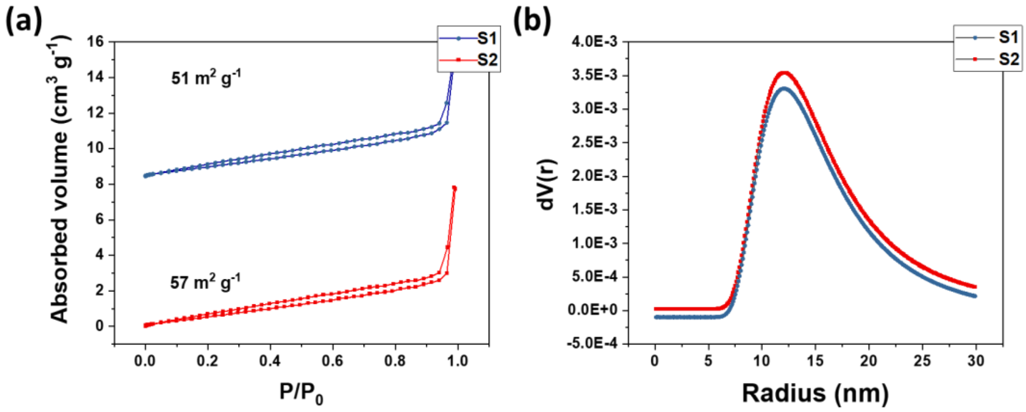

Supplementary Figure 3. N_2 adsorption isotherm experiments. (a) The specific surface areas results. (b) Pore-radius distribution.

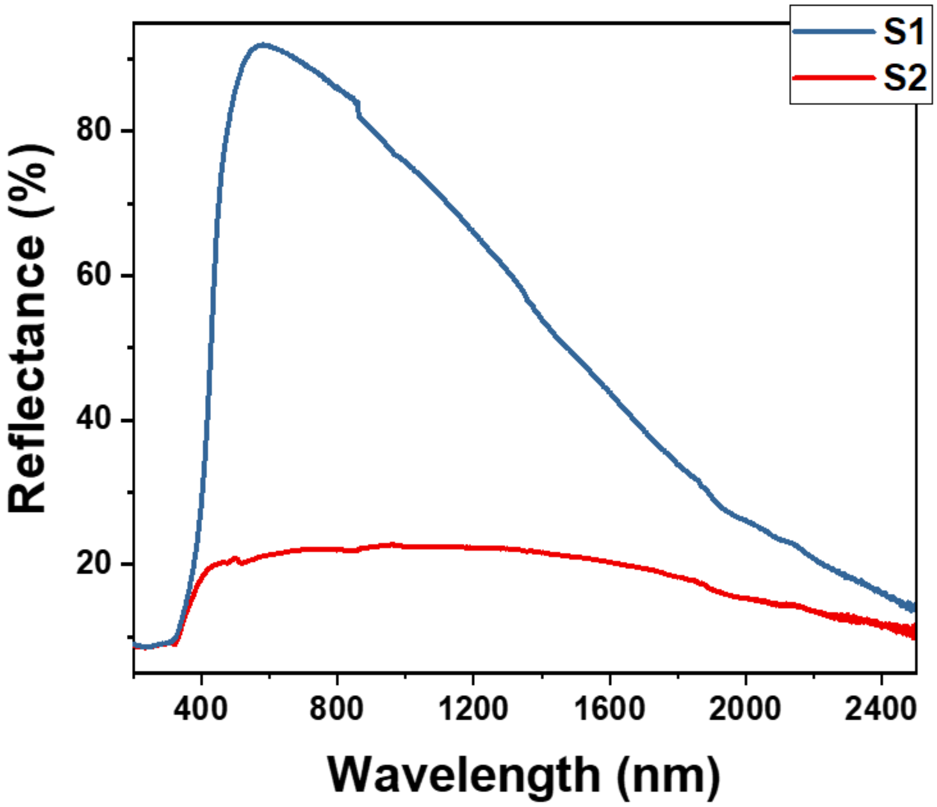

Supplementary Figure 4. Ultraviolet-visible near-infrared spectroscopy. The as-prepared S2 exhibited a greater absorbance than the S1.

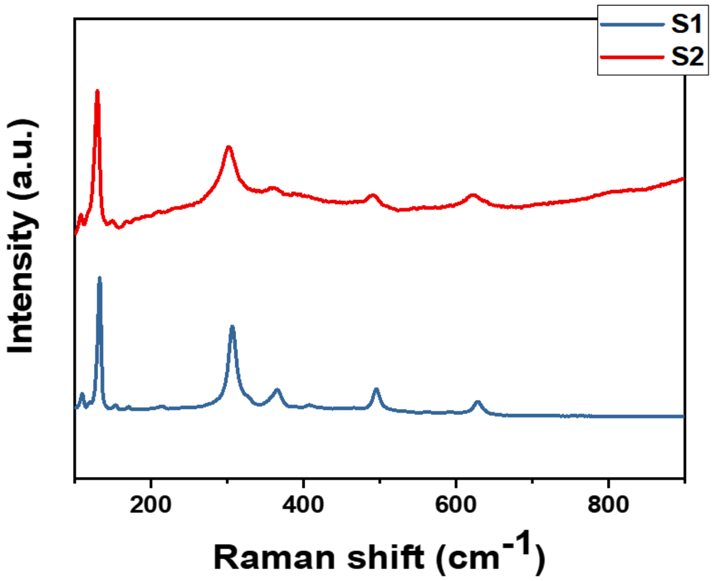

Supplementary Figure 5. The Raman spectra of S1 and S2. Due to the strong phonon confinement induced by the defects, the Raman spectrum of S2 exhibits a redshift, and the broad peaks of S2 represent the amorphization.

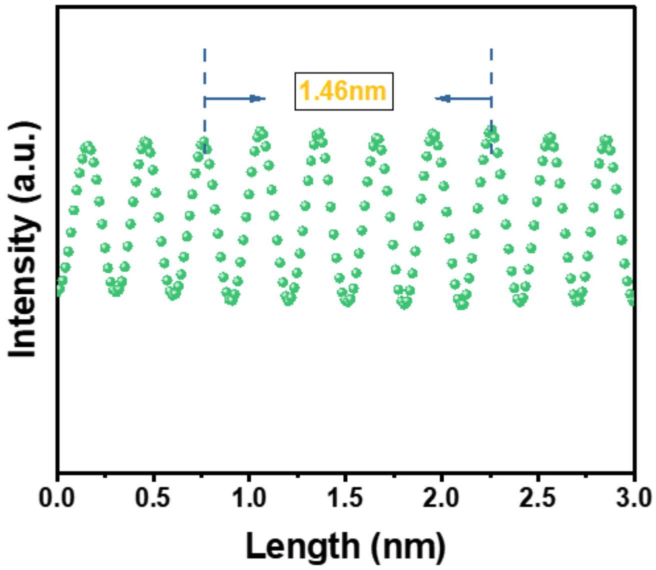

Supplementary Figure 6. The measured lattice constant of S2 (222). The value is calculated as 1.46/5 nm= 0.292 nm.

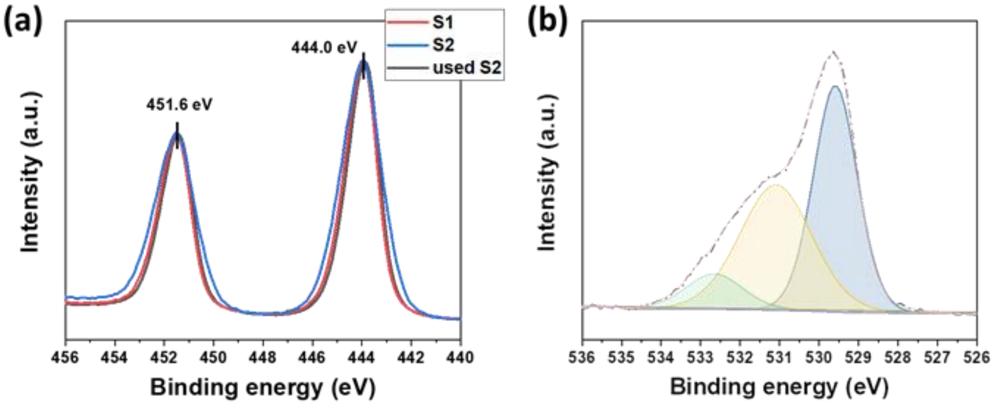

Supplementary Figure 7. XPS spectra of samples. (a) In*3d* spectra, (b) the used S2 O*1s* spectra.

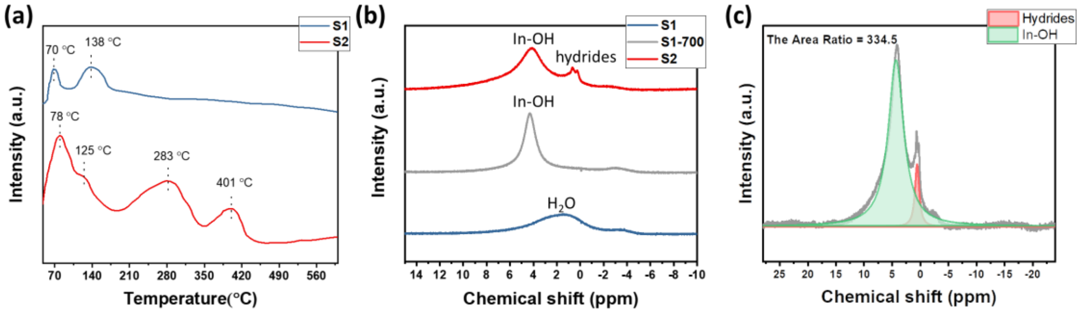

Supplementary Figure 8. Surface analysis of samples. (a) H_2-TPD spectra of S1 and S2. (b) ^1H solid-state MAS NMR spectroscopy for S1, treated (annealing at 700 °C for 5 hours, denoted S1-700) and S2; S1 sample absorbs H_2O from the air and contains mirror OH groups. (c) Fitting the peak of hydride and hydroxyl in the ^1H solid-state MAS NMR spectroscopy of S2. After annealing at 700 °C for 5 hours, surface H_2O was removed; only OH groups at 4.31 ppm can be observed in S1-700 spectra. The hydrides can induce the two new peaks at 0.253 ppm and 0.607 ppm in the infrastructure of the S2 sample. We can conclude that the new black indium oxide can be written as $H_zIn_2O_{3-x}(OH)_y$.

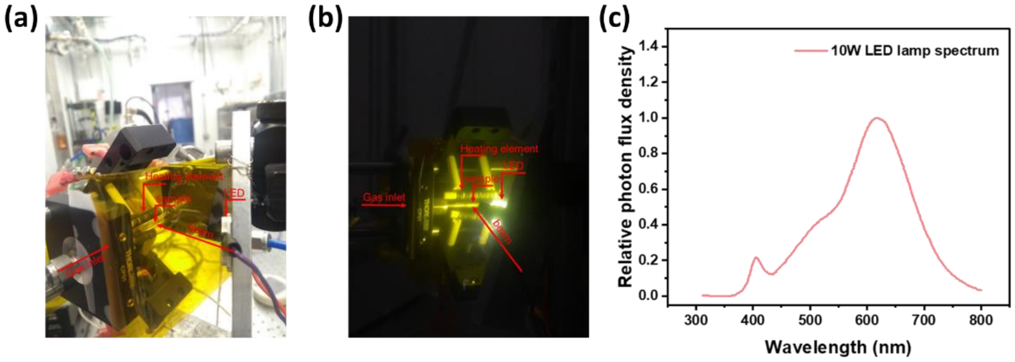

Supplementary Figure 9. In situ XAS setup images. (a-b) The picture of in situ XAS cell. (c) The output spectrum of a 10W LED lamp.

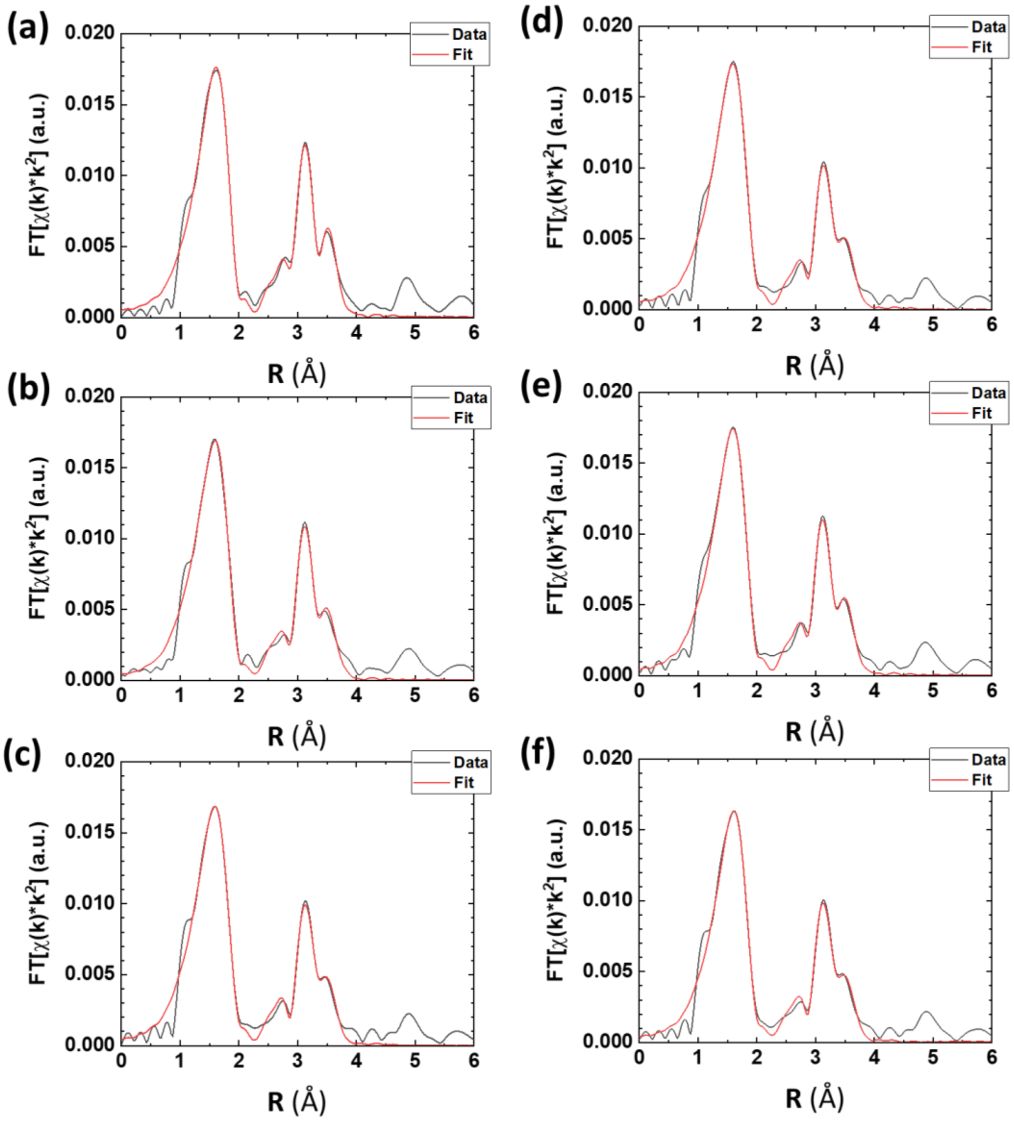

Supplementary Figure 10. Fourier transforms of k^3-weighted EXAFS spectrum. (a) Under the dark conditions at 200 °C, (b) Under the dark conditions at 260 °C, (c) Under the dark conditions at 300 °C, (d) Under the light conditions at 200 °C, (e) Under the light conditions at 260 °C, (f) Under the light conditions at 300 °C.

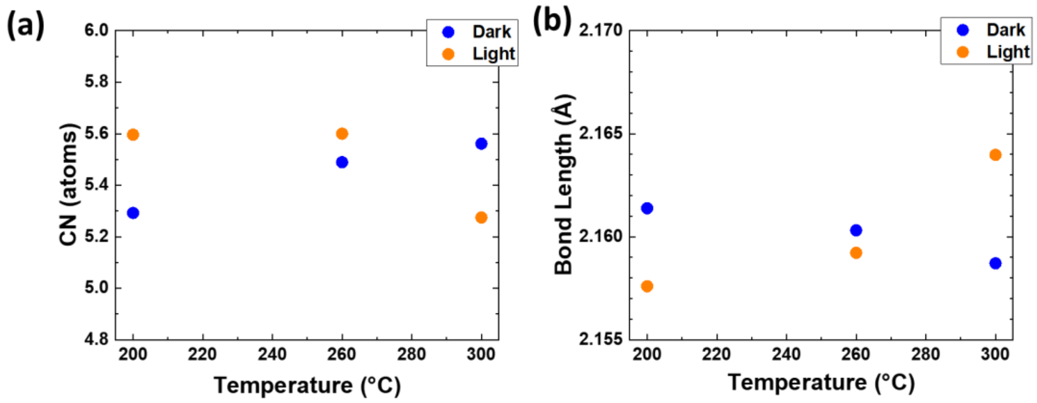

Supplementary Figure 11. ***In-situ* XAS results**. (a) Coordination number, (b) the average bond length.

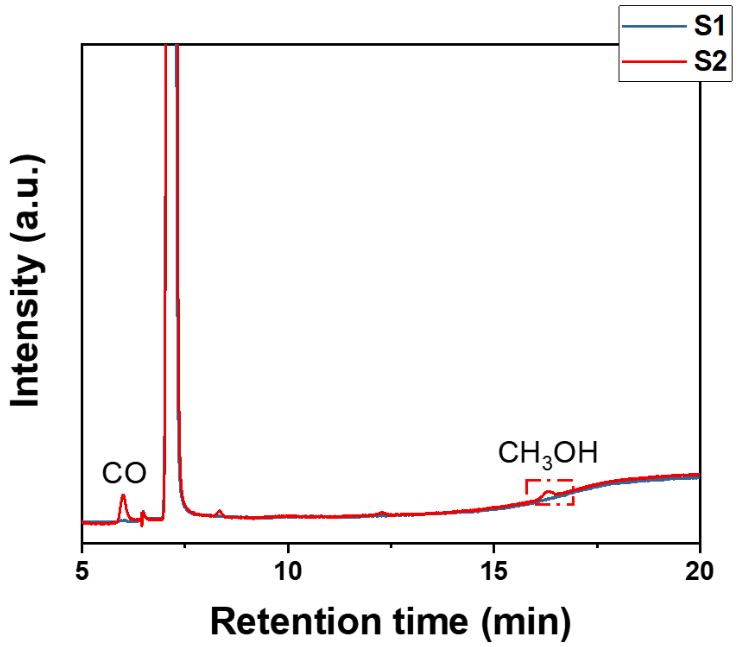

Supplementary Figure 12. The chromatography spectra: signal of S1 (blue line) and S2 (red line).

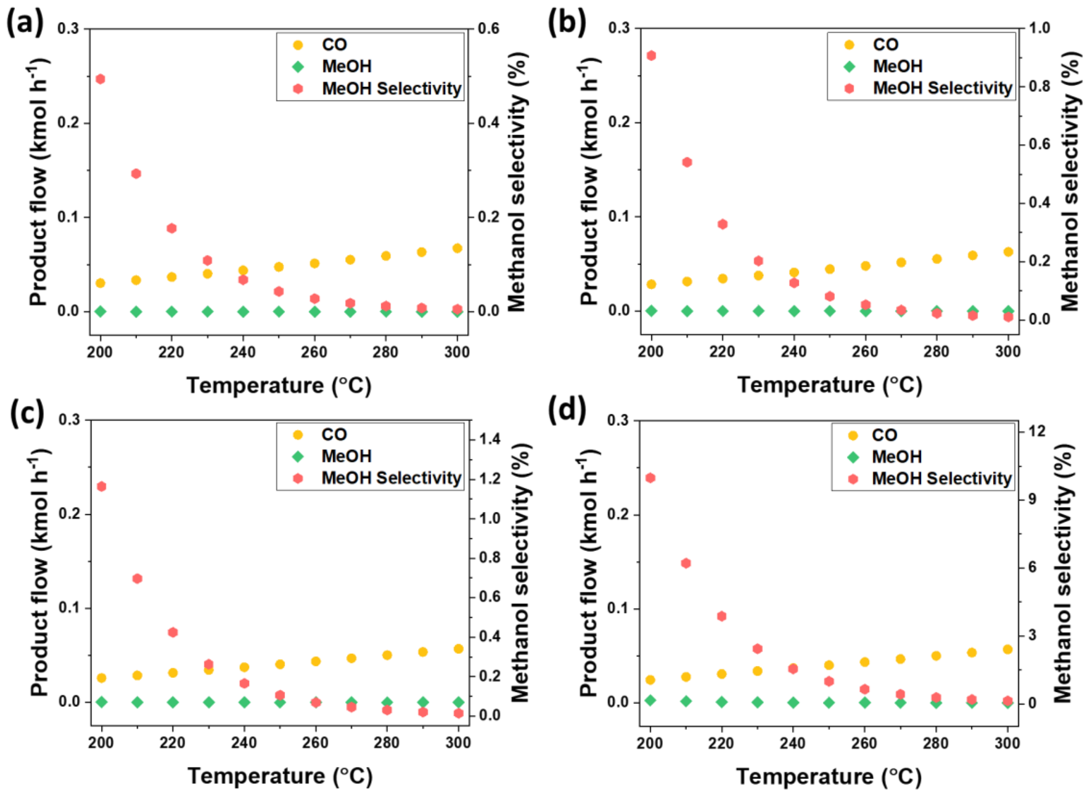

Supplementary Figure 13. Simulated methanol (MeOH) selectivity under thermodynamic equilibrium at atmospheric pressure and with different H_2/CO_2 ratios: (a) 3:1, (b) 2:1, (c) 1:1, and (d) under 30 Psia pressure with H_2/CO_2 ratios = 3:1. The simulation for calculating the methanol equilibrium yields and selectivities used the Gibbs reactor block in Aspen Plus V11 with the ideal NRTL property package. The specified reactions were RWGS and methanol synthesis.

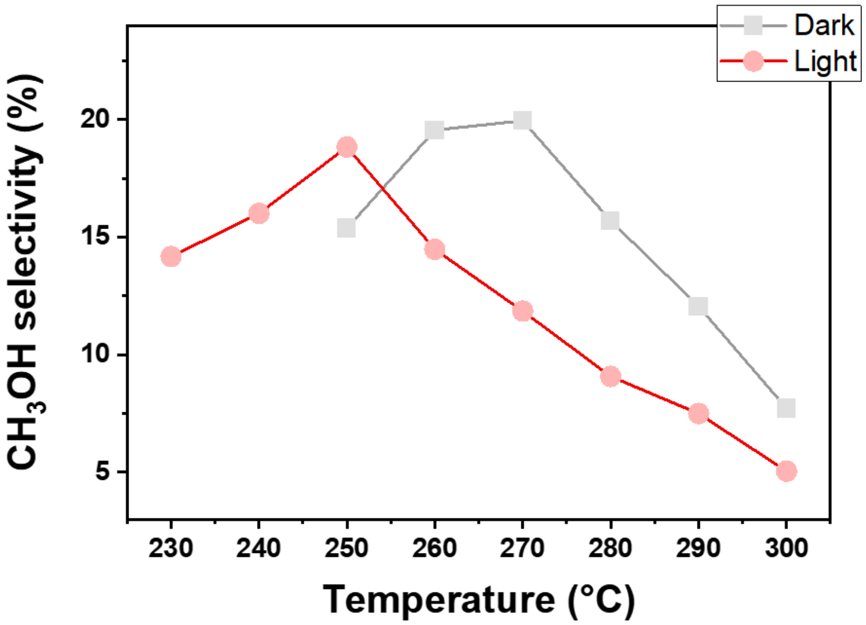

Supplementary Figure 14. The initial methanol selectivity over S2. Under CO + H_2 (1:1) mixed gas with the dark/light conditions.

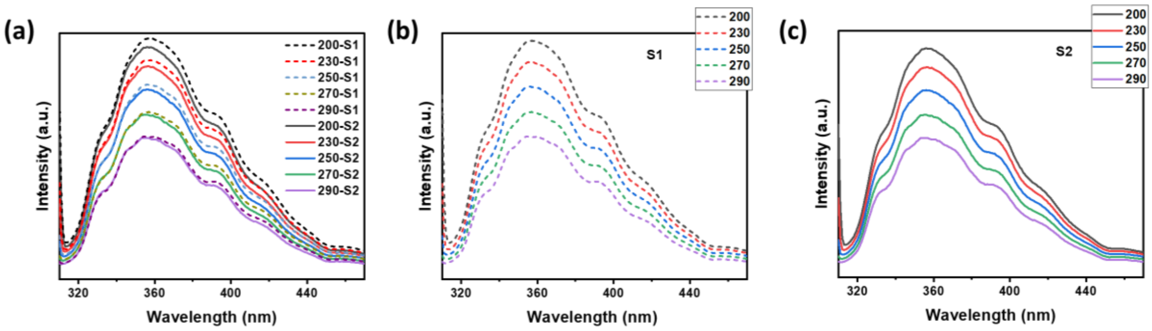

Supplementary Figure 15. The temperature programmed photoluminescence spectra of samples. (a) Summary photoluminescence spectra of S1 and S2 at different temperatures (200–290 °C). Separate PL spectra of (b) S1 and (c) S2.

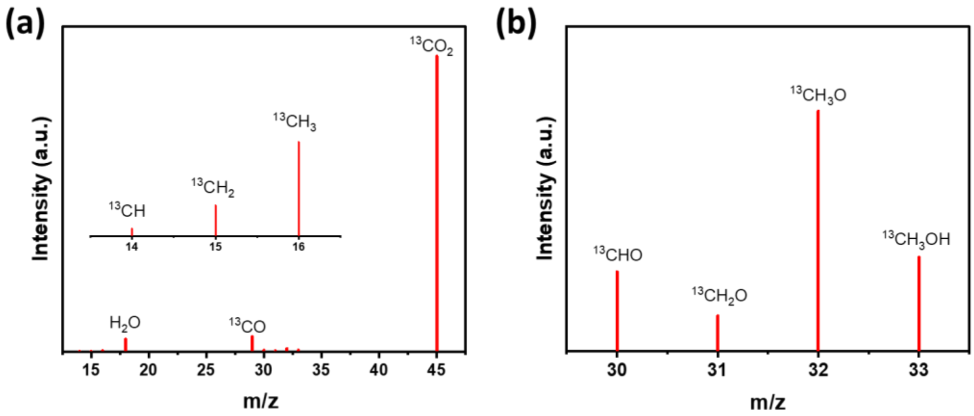

Supplementary Figure 16. The isotope-labeled $^{13}CO_2$ experiment. (a) Representative mass spectra with the m/z in the range 13-45. (b) The CH_3OH products when using the isotope-labeled $^{13}CO_2$ feedstock.

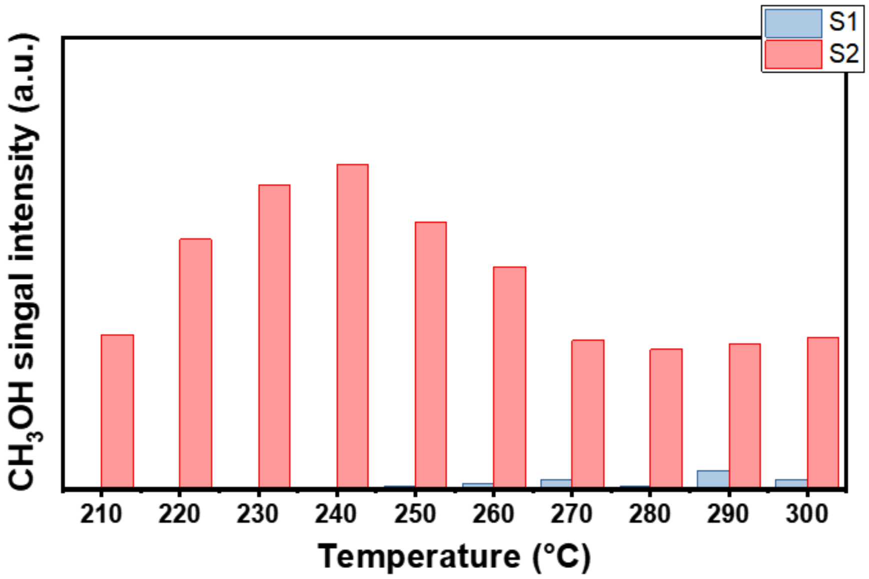

Supplementary Figure 17. Catalytic performance towards CO hydrogenation. CO hydrogenation test was conducted under CO + H_2 (1:1) with methanol signal (m/z = 31). These results confirmed that CO can be used as the feedstock for methanol synthesis.

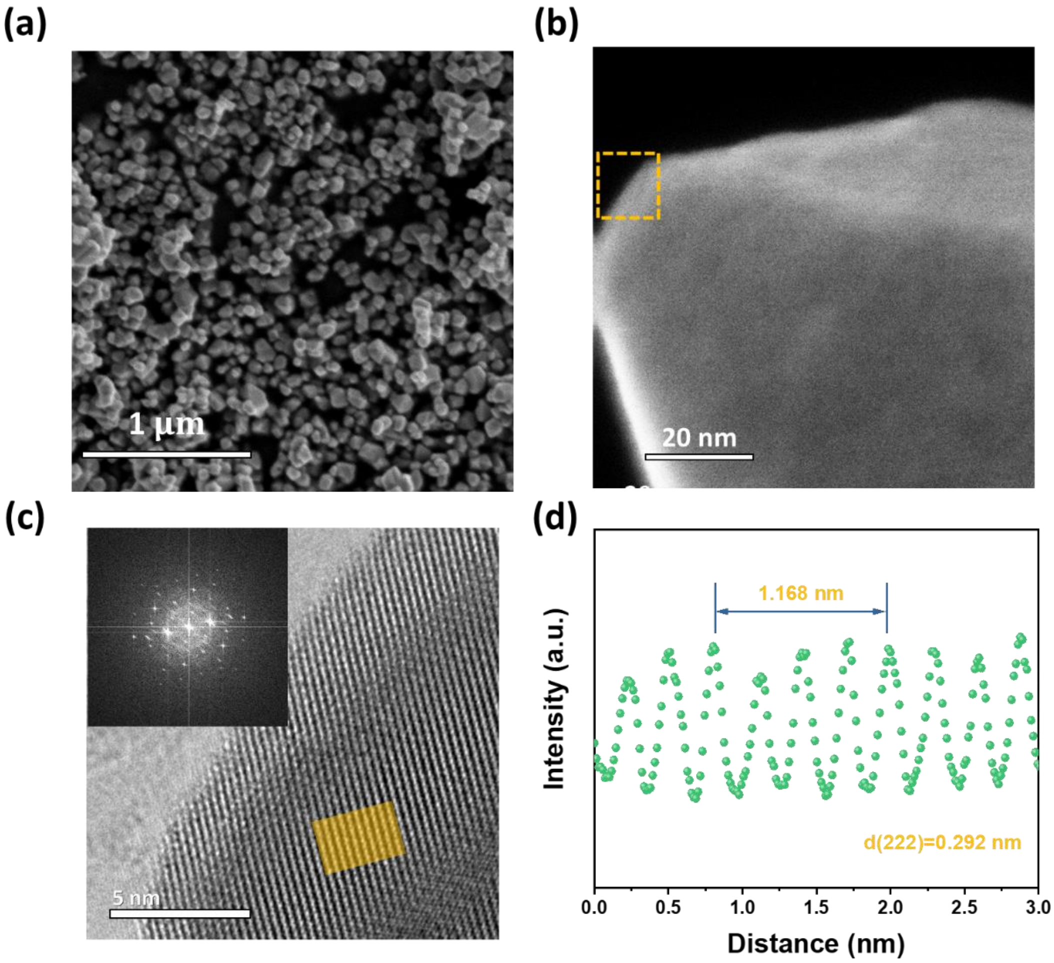

Supplementary Figure 18. Structural characterizations of the used S2. (a) The SEM image of the used S2, (b–c) the TEM image of the used S2, and (d) the corresponding d-spacing e of the used S2.

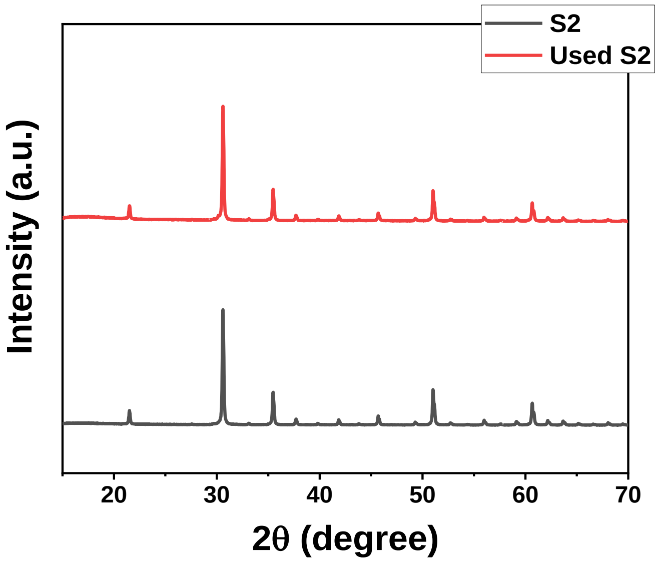

Supplementary Figure 19. Powder XRD patterns of used S2.

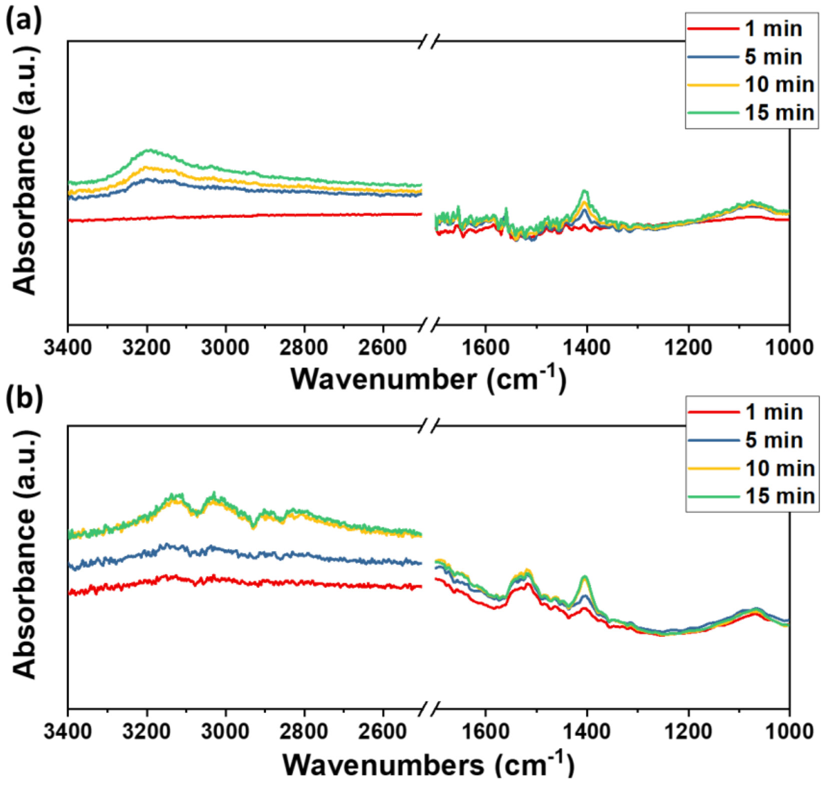

Supplementary Figure 20. ***In-situ* DRIFTS of S2**. (a) CO_2 and H_2, and (b) CO and H_2 at 300 °C. 3200 cm^{-1} can be attributed to the HCOOH of O-H stretching; 2800–3000 cm^{-1} and 1400cm^{-1} can be attributed to the H_3COH or H_3CO of C-H stretching; 1520 cm^{-1} and 1370 cm^{-1} are assigned to the asymmetric and symmetric OCO stretching vibrations,[1, 2] respectively, of adsorbed bidentate HOCO* species. The peaks at 2931 cm^{-1}, 2824 cm^{-1}, and 1046 cm^{-1} are attributed to the H_3CO* species of O-H stretching.[3] 1649 cm^{-1} can be attributed to the H_2O. In **Figure3b** and **Supplementary Figure 20b**, S2 corresponds to the *in-situ* DRIFTS, indicating that CO hydrogenation is easier towards methanol on the S2 surface, and the catalytic reaction temperature is lower than that of CO_2 hydrogenation.

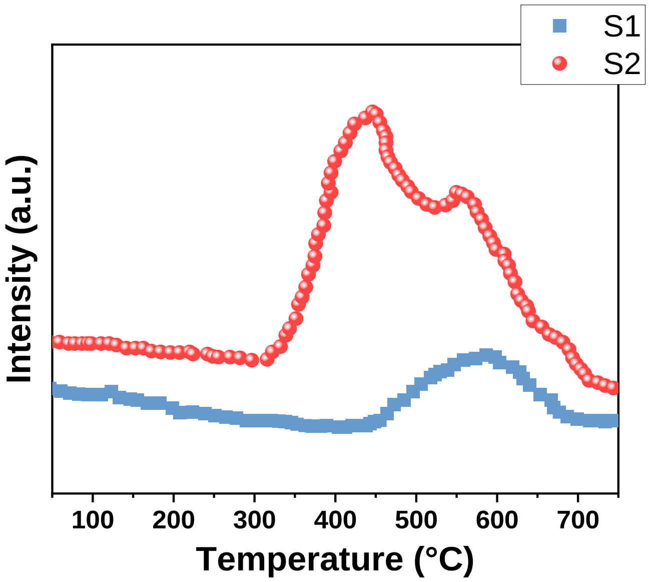

Supplementary Figure 21. CO-TPD spectra of S1 and S2.

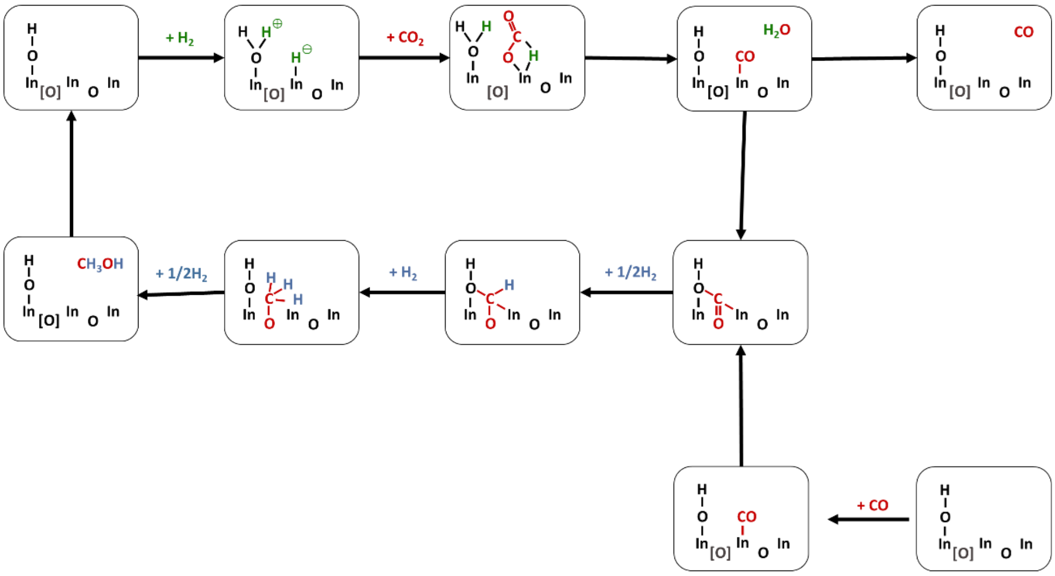

Supplementary Figure 22. Proposed catalytic pathway for CO_2 hydrogenation over black Indium oxide.

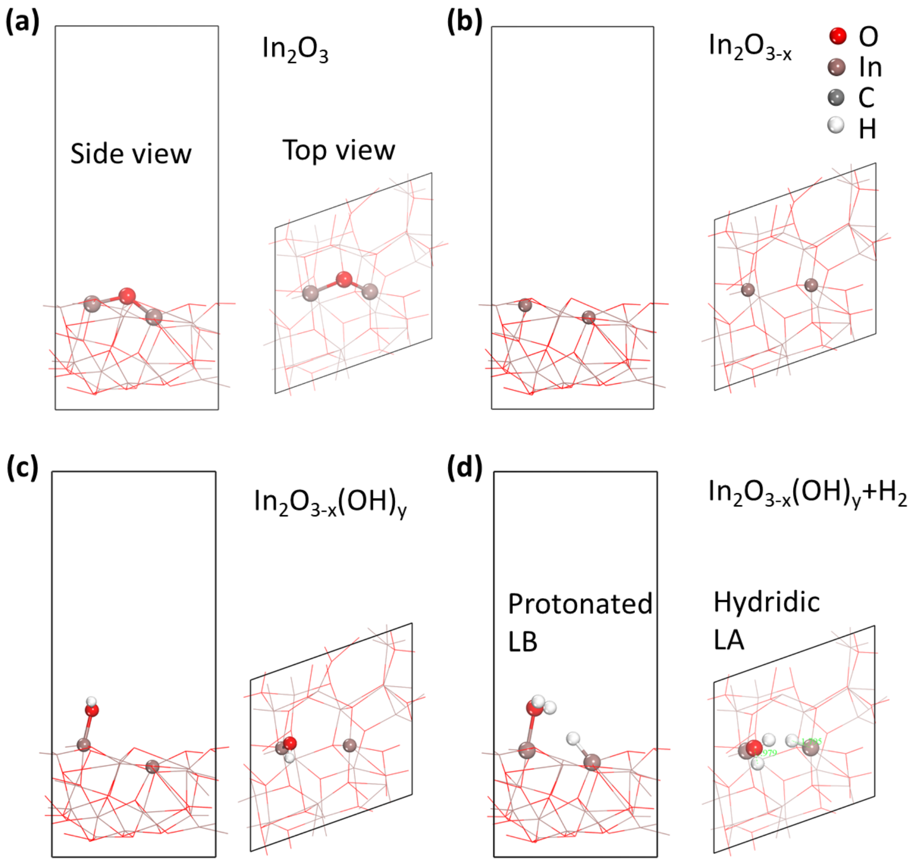

Supplementary Figure 23. DFT models. (a) In_2O_3, (b) In_2O_{3-x}, (c) $In_2O_{3-x}(OH)_y$, and (d) surface FLPs of $In_2O_{3-x}(OH)_y$ after H_2 heterolysis.

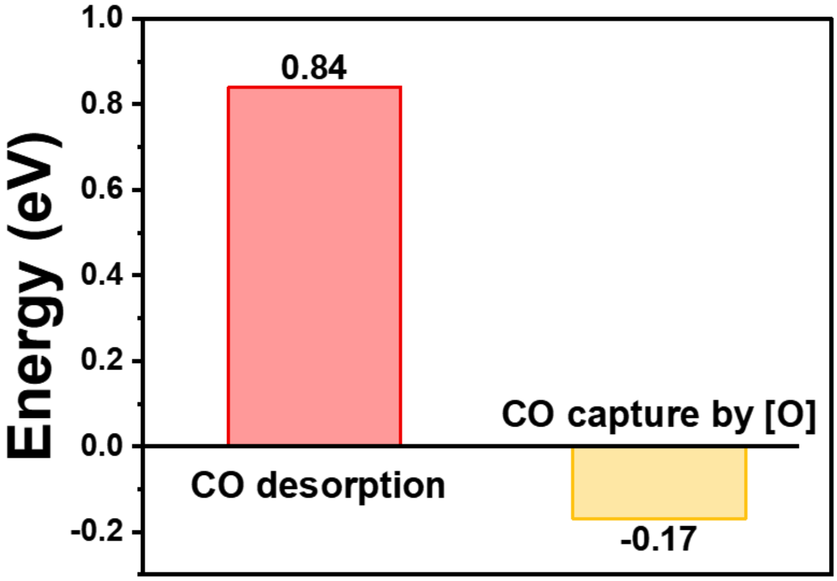

Supplementary Figure 24. Energy diagram of CO desorption and CO capture by [O].

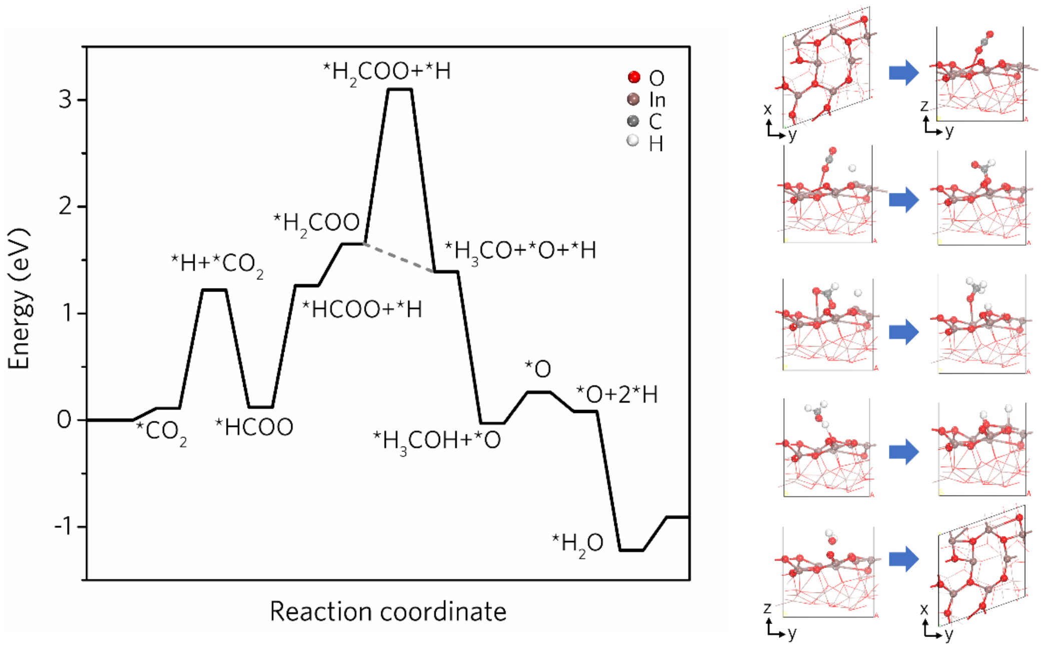

Supplementary Figure 25. Energy diagram of methanol synthesis. Methanol synthesis over [O] of In_2O_{3-x} via a "CO_2-H_zCO_2-CH_3OH" (z = 1, 2 and 3) pathway (left) and corresponding configurations against the reaction coordinate (right). The gray dash line represents the possible molecular hydrogenation pathway while the black line represents the stepwise hydrogenation via atomic H.

Supplementary References

1 Yan, T. J. et al. Polymorph selection towards photocatalytic gaseous CO_2 hydrogenation. *Nat. commun*. **10**, 2521 (2019).

2 Kunkes, E. L. et al. Hydrogenation of CO_2 to methanol and CO on Cu/ZnO/Al_2O_3: Is there a common intermediate or not? *J. Catal.* **328**, 43-48 (2015).

3 Kattel, S. et al. Tuning Selectivity of CO_2 Hydrogenation Reactions at the Metal/Oxide Interface. *J. Am. Chem. Soc.* **139**, 9739-9754 (2017).
