# Supplementary figures and images for: New black indium oxide—tandem photothermal CO2-H2 methanol selective catalyst
Source: Nat Commun. 2022 Mar 21;13:1512. doi: 10.1038/s41467-022-29222-7 (PMC8938479; doi:10.1038/s41467-022-29222-7)

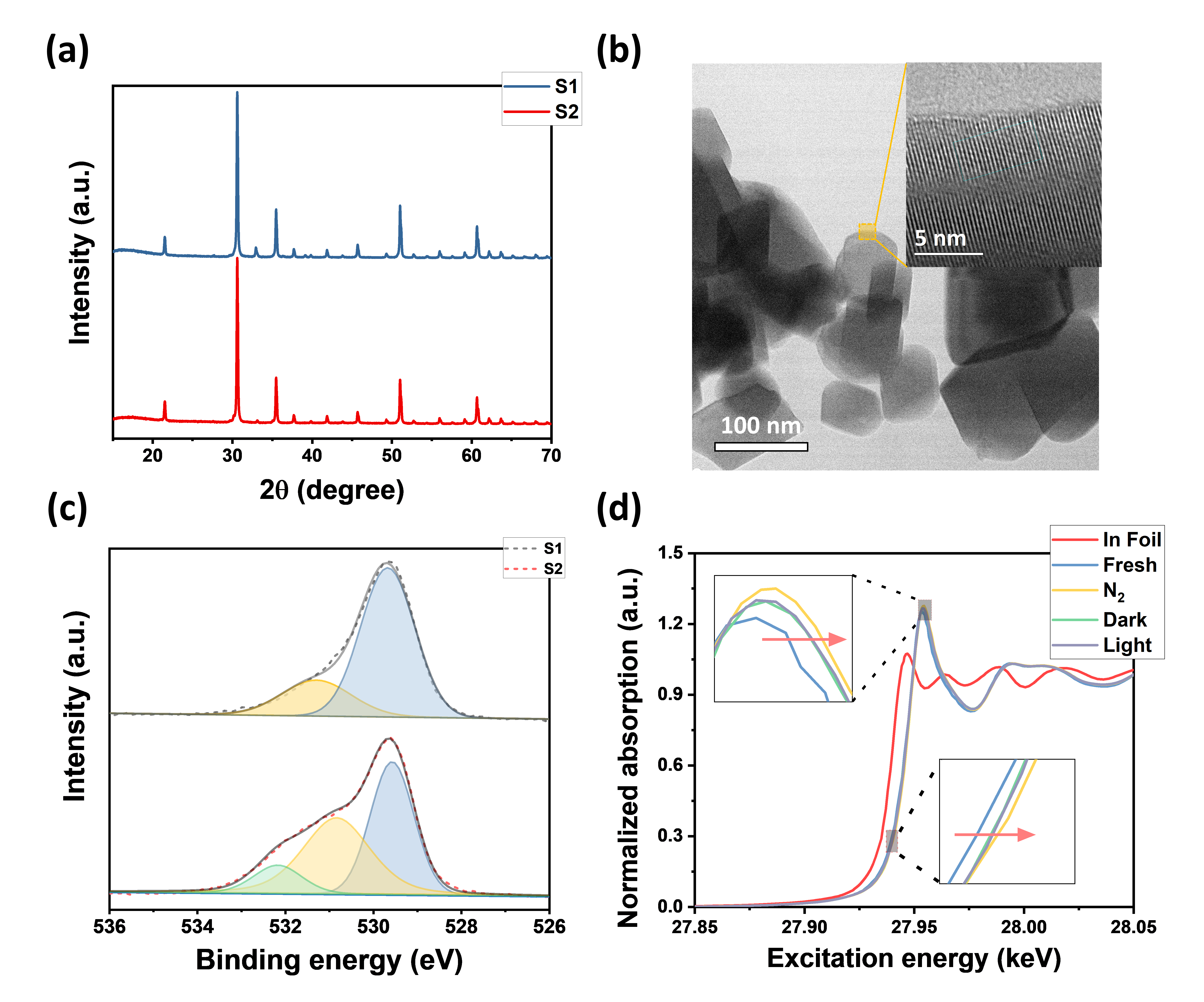

Supplement: Supplementary file 3 — Source Data [file 41467_2022_29222_MOESM3_ESM.zip › Figures/figure 1-4/Figure 1.TIF]

## Slide 1
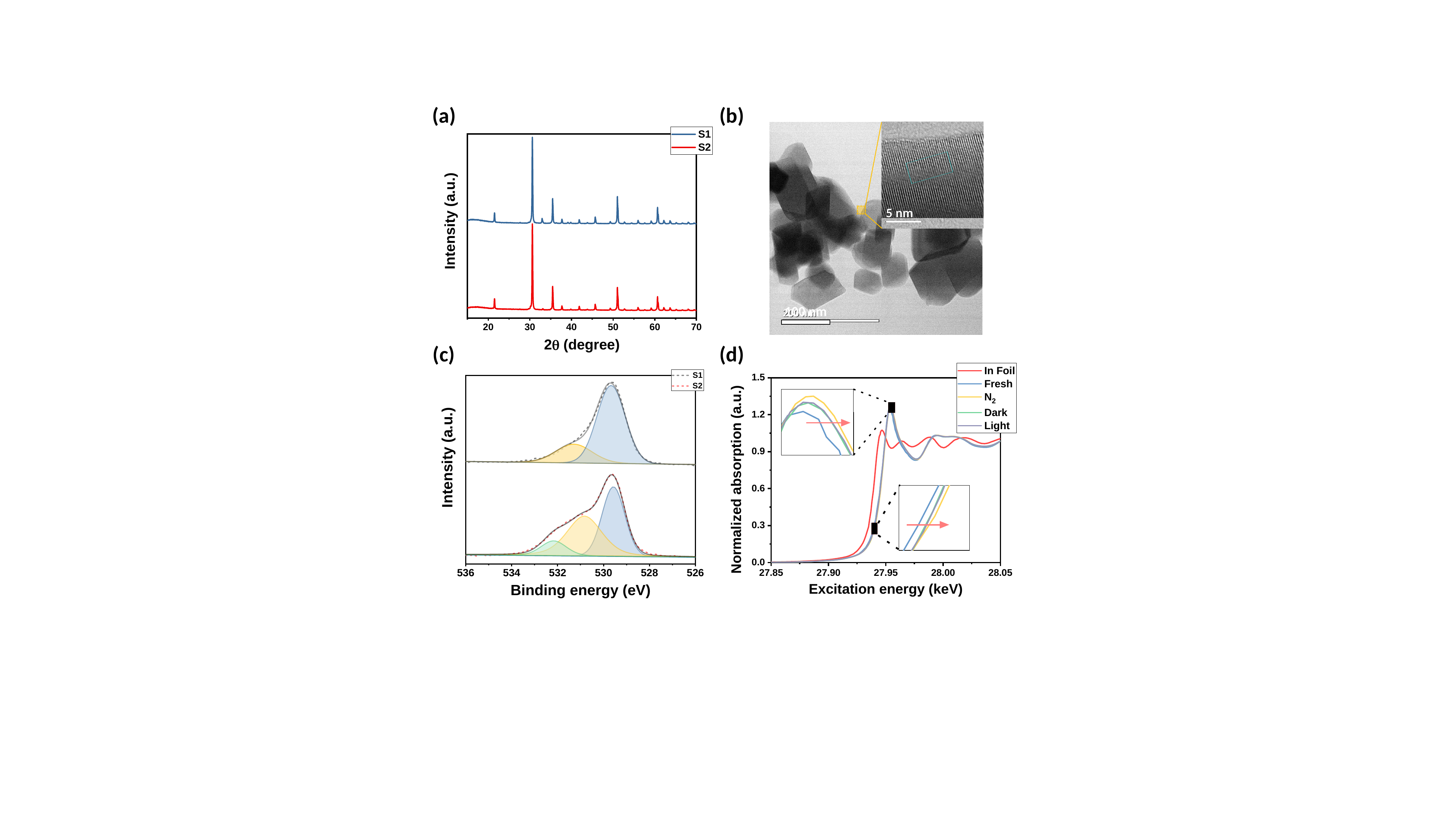

(a)
(b)
(c)
(d)
100 nm

## Slide 2
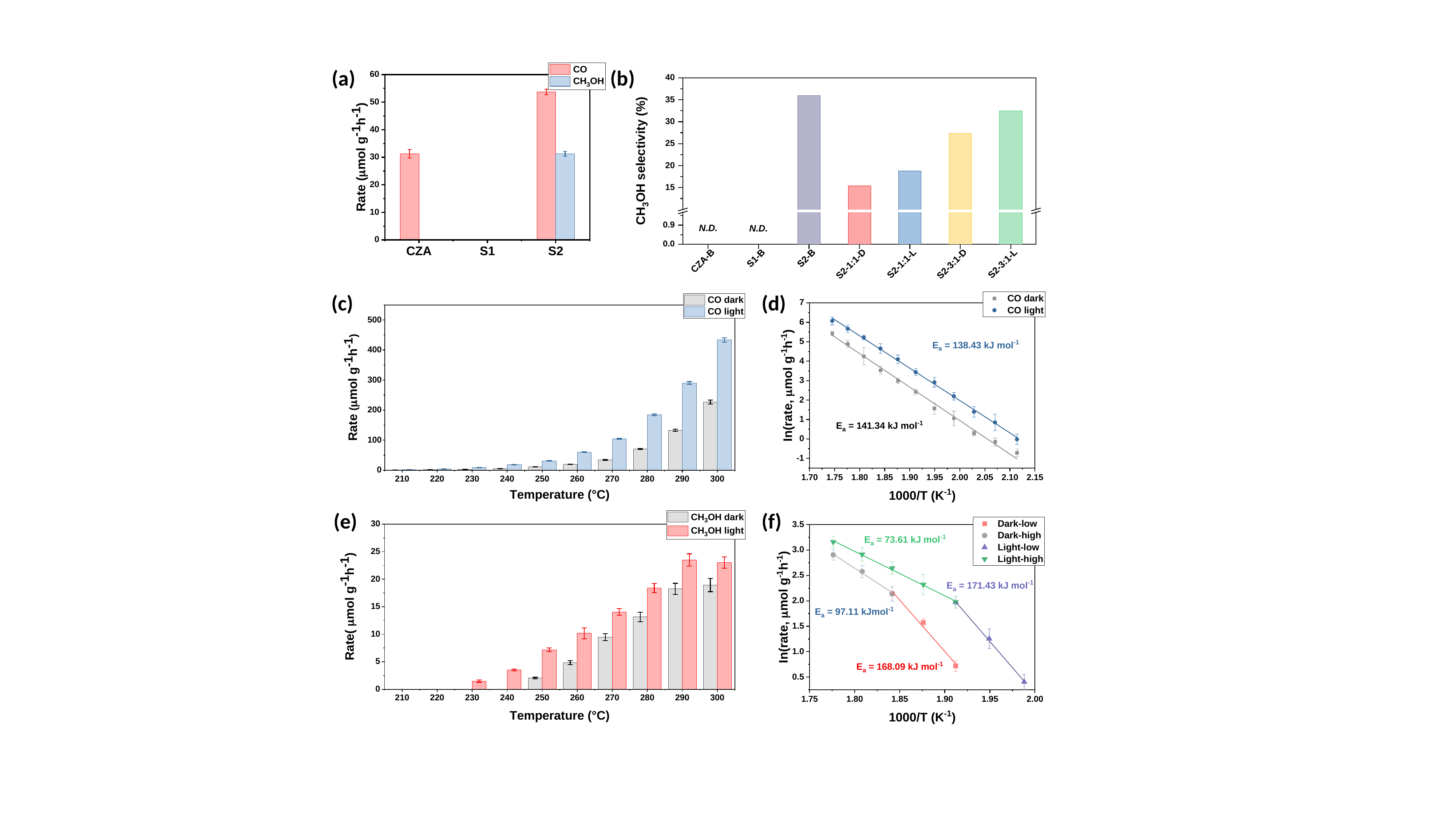

(a)
(b)
(c)
(d)
(e)
(f)

## Slide 3
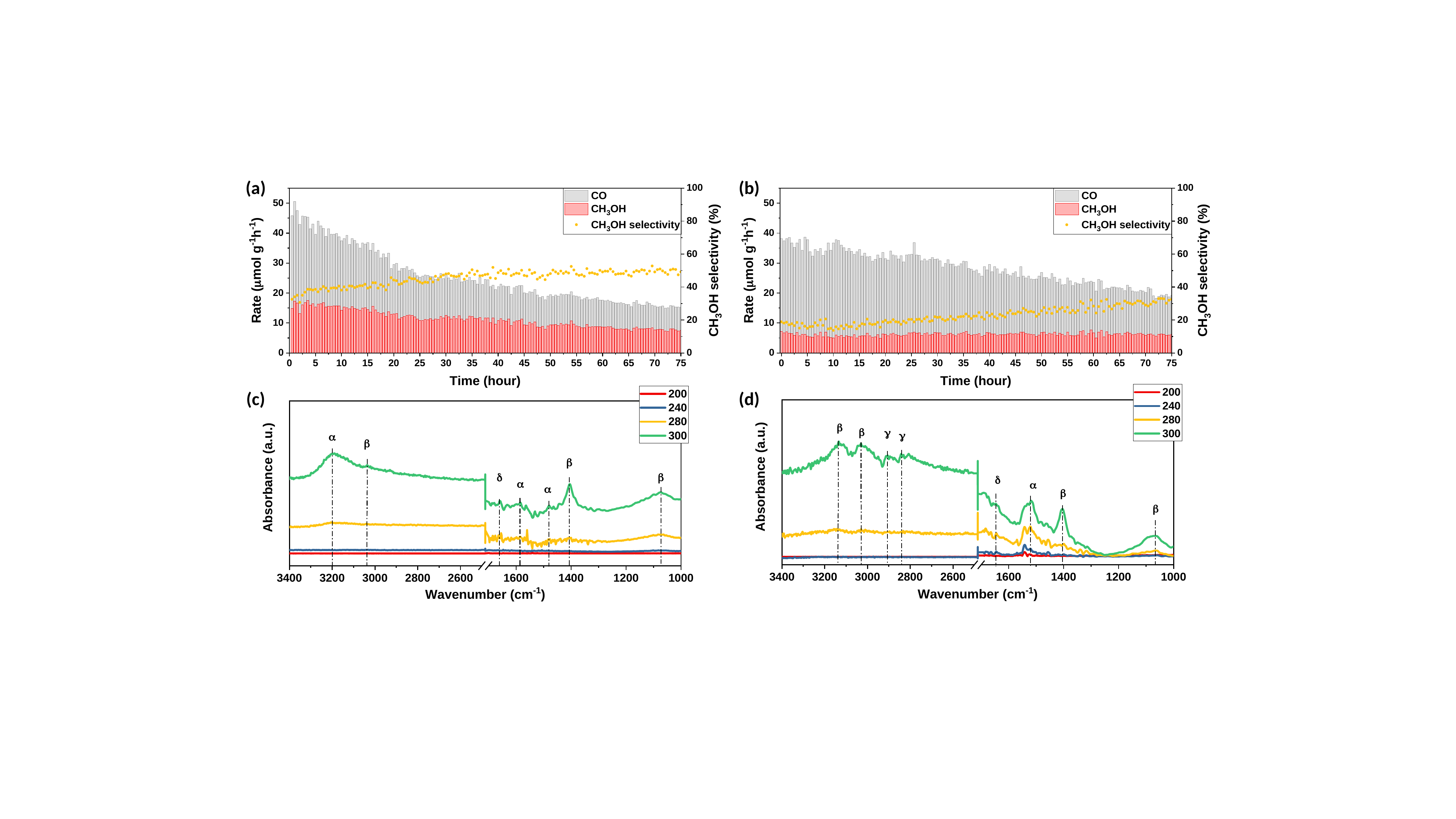

(a)
(b)
(c)
(d)

## Slide 4
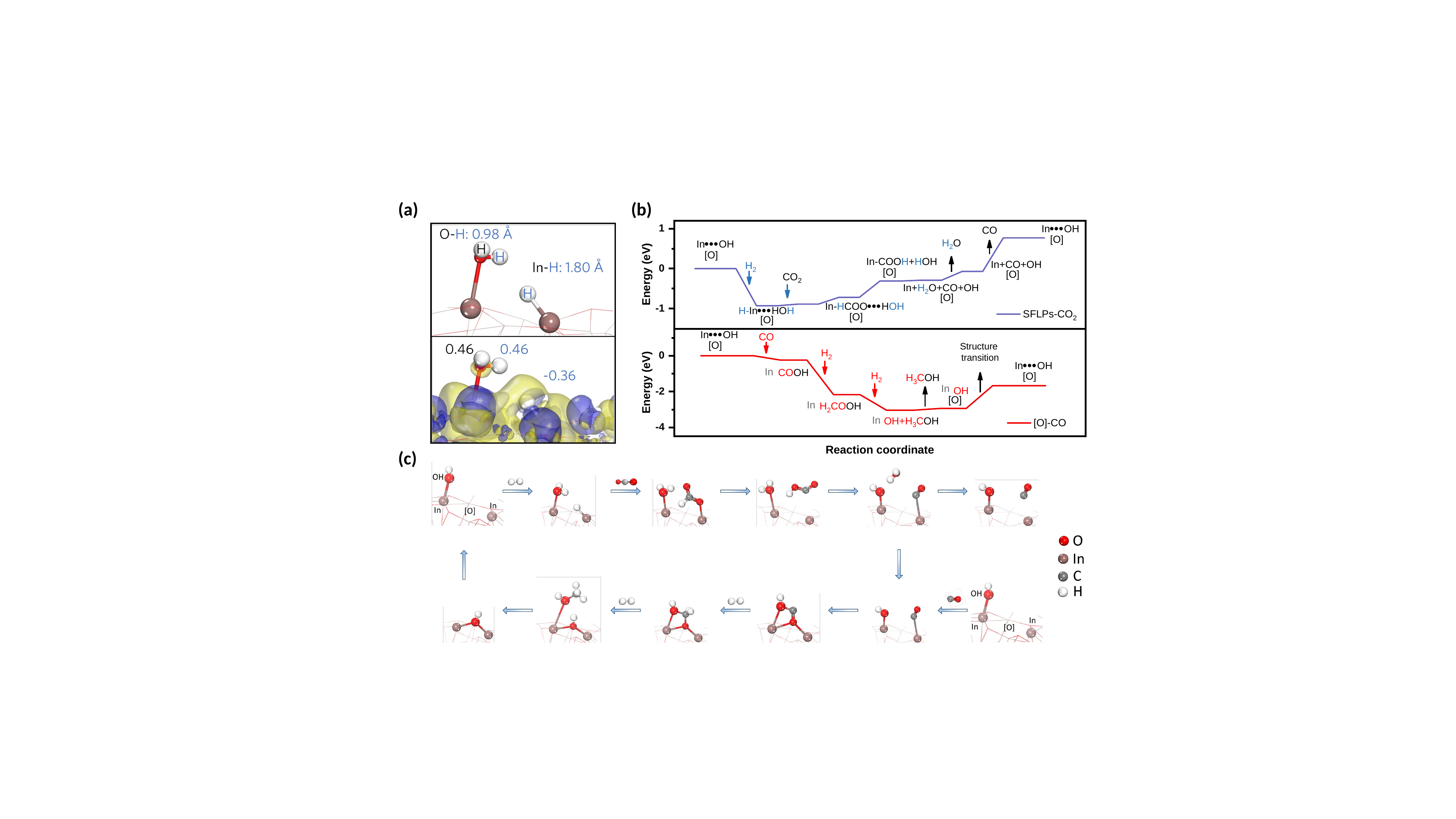

(a)
(b)
(c)

Supplement: Supplementary file 3 — Source Data [file 41467_2022_29222_MOESM3_ESM.zip › Figures/figure 1-4/Figure 1-4.pptx]

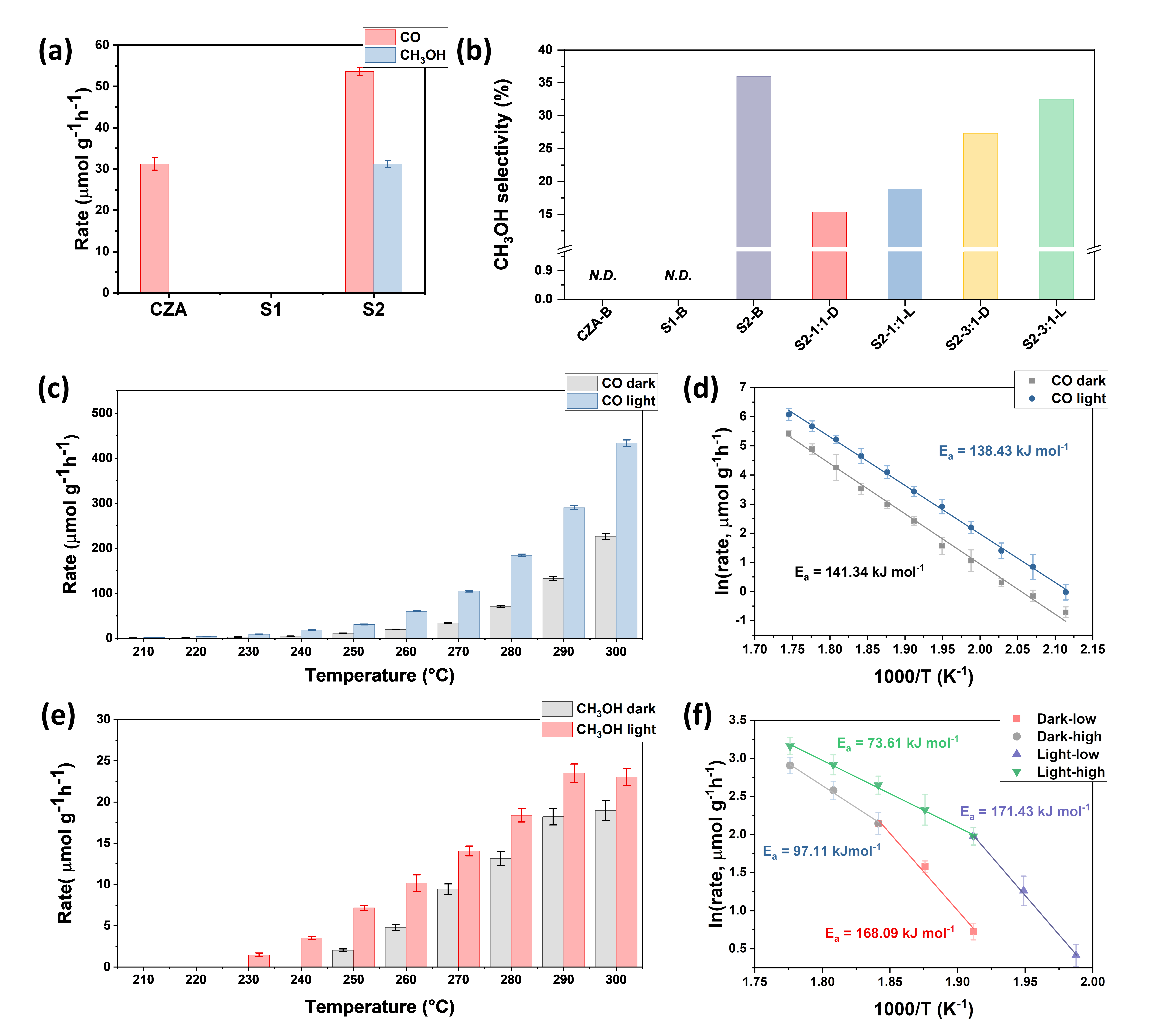

Supplement: Supplementary file 3 — Source Data [file 41467_2022_29222_MOESM3_ESM.zip › Figures/figure 1-4/Figure 2.TIF]

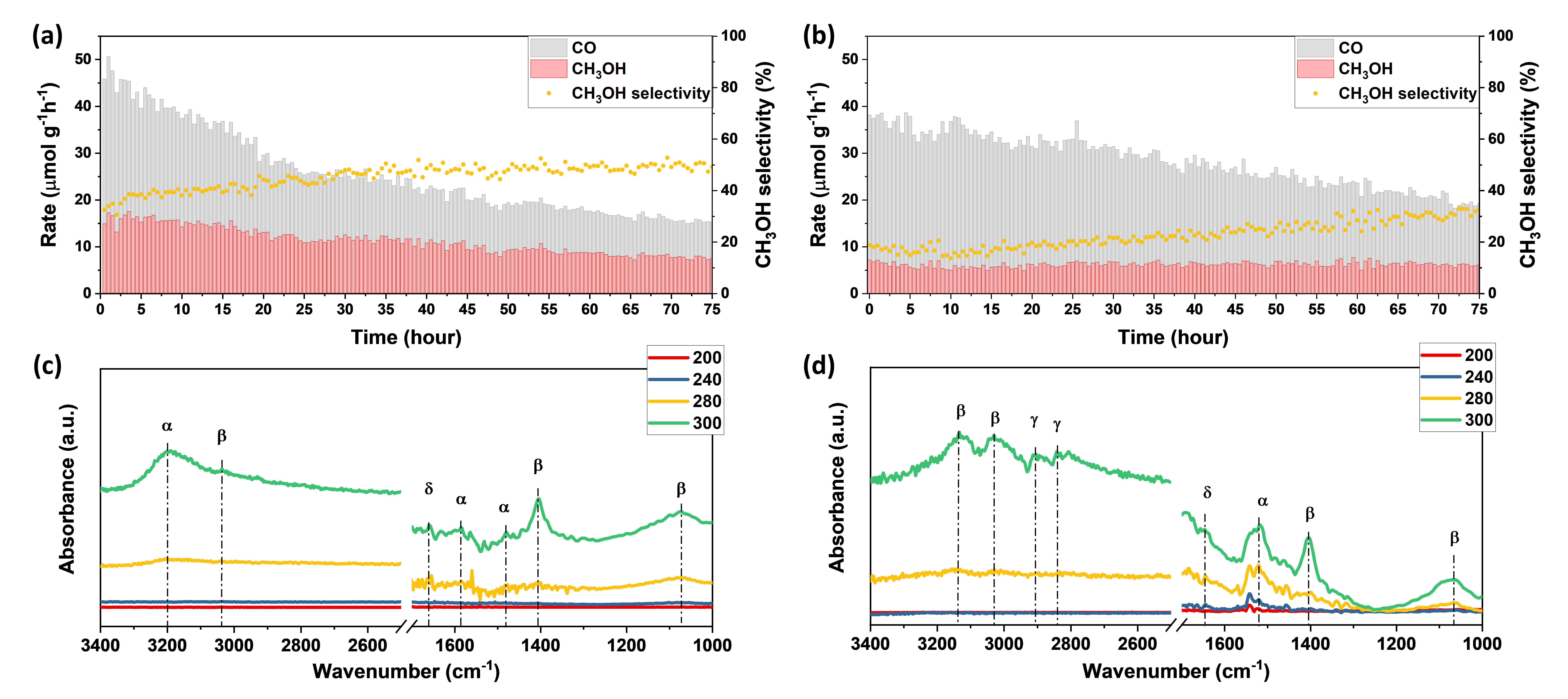

Supplement: Supplementary file 3 — Source Data [file 41467_2022_29222_MOESM3_ESM.zip › Figures/figure 1-4/Figure 3.TIF]

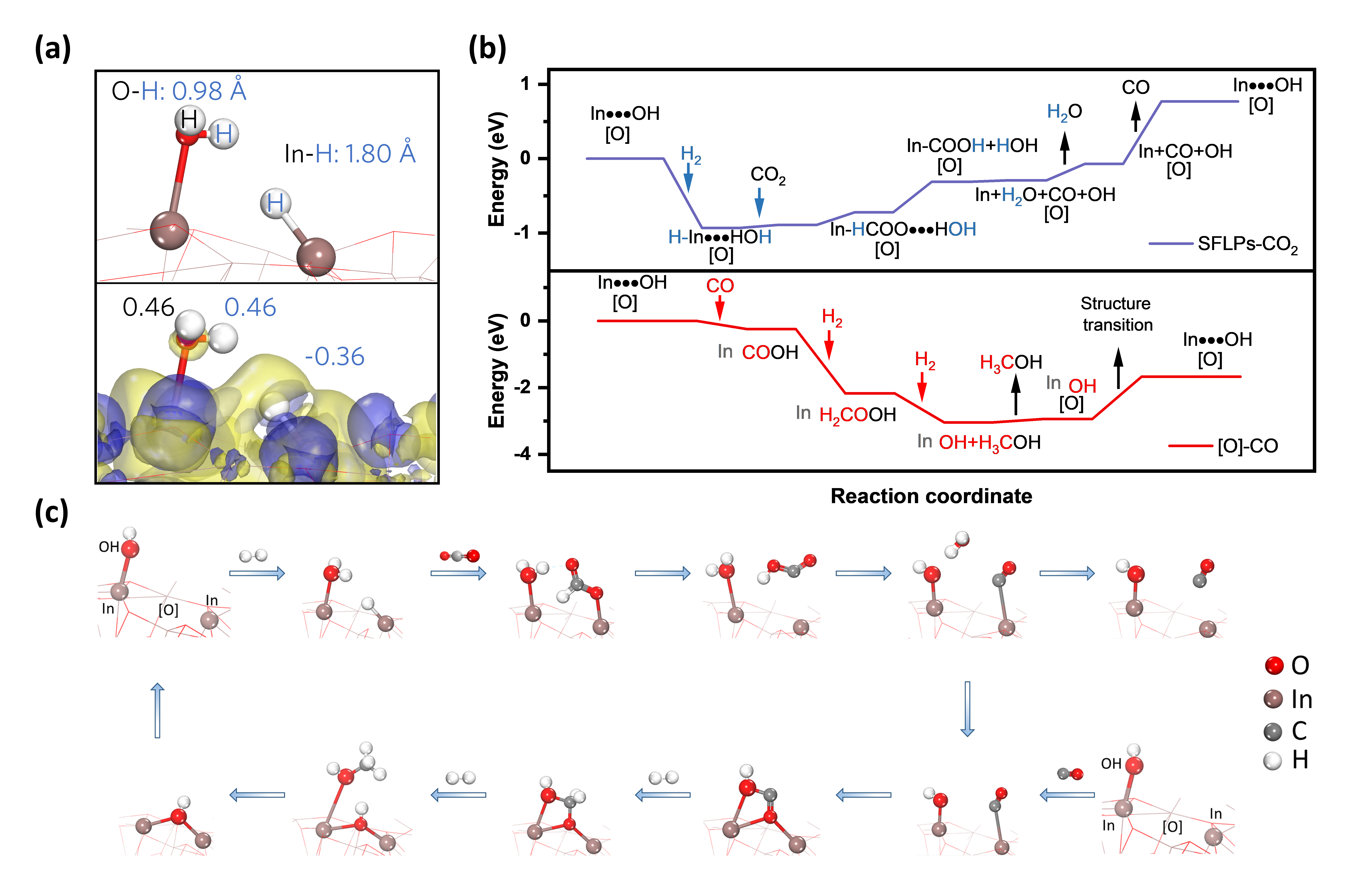

Supplement: Supplementary file 3 — Source Data [file 41467_2022_29222_MOESM3_ESM.zip › Figures/figure 1-4/Figure 4.tif]
